# Glucose Transporter 1 (GLUT1) Positivity in Ganglion Cysts: A Diagnostic Caveat Due to Overlap With Perineuriomas and Other Fibromyxoid Lesions

**DOI:** 10.7759/cureus.77142

**Published:** 2025-01-08

**Authors:** Ahmed Lazim, Faizan Malik, Kumarasen Cooper

**Affiliations:** 1 Pathology, Temple University Hospital, Philadelphia, USA; 2 Anatomic Pathology, St. Jude Children's Research Hospital, Memphis, USA; 3 Pathology, Hospital of the University of Pennsylvania, Philadelphia, USA

**Keywords:** core biopsy, ema, ganglion cyst, glut-1, perineurioma

## Abstract

Glucose transporter 1 (GLUT1) is a sensitive immunohistochemical marker for perineural cells and certain mesenchymal neoplasms, including chordoma, desmoplastic small round cell tumor, desmoid-type fibromatosis, epithelioid sarcoma, gastrointestinal stromal tumor, myoepithelioma, schwannoma, and undifferentiated pleomorphic sarcoma. In this study, we focus on the morphological and immunohistochemical challenges in differentiating between ganglion cysts and perineuriomas. To our knowledge, no previous studies have explored the potential morphological and immunohistochemical similarities between these two lesions. We retrospectively retrieved cases coded as “ganglion cysts” from our departmental archives. Clinical data included the patient’s age, gender, lesion location, and size. All slides were reviewed by two pathologists, and 4-μm-thick formalin-fixed paraffin-embedded tissue sections were subjected to immunostaining for epithelial membrane antigen (EMA) and GLUT1. The study included core biopsies and excision specimens from 14 patients (11 females and three males; M:F ratio: 3.6:1), ranging in age from 20 to 89 years (median: 60 years; mean: 53 years). Lesion locations included the finger (six cases), dorsal wrist (five cases), and the knee, volar wrist, and an unspecified hand site (one case each). Morphologically, 11 ganglion cysts were predominantly cystic, with walls composed of dense fibroconnective tissue and focal myxoid changes. These myxoid areas contained spindled and stellate cells within a myxoid stroma. The remaining three cases exhibited a predominantly solid morphology, characterized by dense fibroblastic-myofibroblastic stroma with limited myxoid degeneration and small spindled and bipolar cells. Scant chronic inflammation was observed in most cases. GLUT1 immunostaining showed membranous patterns in all cases, with some cytoplasmic expression, particularly in stellate cells within the myxoid matrix. EMA staining exhibited focal membranous expression in 12 cases (85.71%) and was negative in two cases (14.29%). These findings highlight the importance of caution when evaluating core biopsies of soft tissue lesions with bland cystic and fibromyxoid histology. The morphological and immunohistochemical similarities between ganglion cysts and perineuriomas may pose a diagnostic challenge and risk of misinterpretation. Awareness of these overlaps is crucial to avoid potential pitfalls in diagnosis.

## Introduction

Perineurial cells are modified fibroblasts that form a protective sheath around nerve fascicles, playing an integral role in the structure and function of peripheral nerves. Ultrastructurally, these cells are characterized by scattered intermediate filaments, a basal lamina, and pinocytotic vesicles. Histologically, perineurial cells can be distinguished from Schwann cells by their bipolar, elongated cytoplasmic processes, immunohistochemical expression of epithelial membrane antigen (EMA), and absence of S-100 positivity. Additionally, perineurial cells express the erythrocyte glucose transporter 1 (GLUT1), a marker initially identified in infantile hemangioma.

Soft tissue perineurioma, a peripheral nerve sheath tumor originating from perineurial cells, typically exhibits an EMA+/S-100-/GLUT1+ immunophenotype. In the appropriate morphological context, GLUT1 expression serves as a valuable diagnostic marker for perineuriomas [1-4].

However, GLUT1 expression is not entirely specific for perineurial differentiation. Ahrens et al., in a study of 247 bone and soft tissue tumors, demonstrated GLUT1 positivity in a range of mesenchymal neoplasms beyond perineuriomas [5]. Similarly, other studies have documented GLUT1 expression in various mesenchymal tumors, including those unrelated to infantile hemangiomas. Whether this expression reflects lineage-specific features or results from upregulation in hypoxic zones, as suggested by Ahrens et al., remains unclear. Nonetheless, GLUT1 expression is widely considered a useful adjunct for diagnosing perineurial tumors [5-7].

In a recent index case, we identified GLUT1 expression in a ganglion cyst of the knee that was initially misclassified as a perineurioma based on biopsy morphology and immunoprofile. This observation prompted us to investigate the prevalence of GLUT1 expression in typical ganglion cysts of the hands and digits to better understand this diagnostic pitfall.

## Materials and methods

This study was approved by the institutional review board. Cases coded as “ganglion cysts” were retrospectively retrieved from the departmental archives over a two-year period. Clinical data, including patient age, gender, lesion location, and size, were collected from the EPIC system.

Core biopsy specimens were obtained under radiological guidance using ultrasound and CT. The specimens were fixed in neutral buffered formalin for six to 10 hours, processed, and embedded in paraffin. Subsequently, 4-μm-thick formalin-fixed paraffin-embedded (FFPE) tissue sections were prepared and stained with the standard H&E stain.

All core biopsies were followed by excision of the lesions. The excision specimens were examined both grossly and microscopically, with findings correlated to the biopsies. Specimens smaller than 2 cm were entirely submitted for microscopic examination, while larger specimens were extensively sampled. The same fixation and staining protocols were applied to these samples.

In addition to H&E-stained sections, FFPE tissue sections (4-μm thick) were deparaffinized using xylene and rehydrated with ethanol in varying concentrations. Staining protocols included antigen retrieval, application of blocking agents, and the addition of primary and secondary antibodies for immunostaining with EMA (Clone E29, DakoCytomation, HUP) and GLUT1 (SLC2A1, Clone EP141, EPITOMICS, HUP).

Glass H&E-stained slides from all cases were independently reviewed by two pathologists (FM and KC). The immunohistochemistry slides were also reviewed by two pathologists (AL and KC) to assess the staining pattern (membranous versus cytoplasmic) and intensity (weak versus strong).

## Results

Routine H&E slides and immunohistochemical stains for GLUT1 and EMA were examined in core biopsy and excision specimens from 14 patients. The cohort included 11 females and three males (M:F ratio 1:3.6), with ages ranging from 20 to 89 years (median: 60 years; mean: 53 years). The lesion locations were distributed as follows: finger (six cases), dorsal wrist (five cases), and the knee, volar wrist, and an unspecified hand site (one case each).

Morphologically, 11 cases were predominantly cystic, with walls composed of dense fibroconnective tissue and focal myxoid changes. The lesional cells were small, spindled, and stellate, set within a background of loose, abundant myxoid stroma. The remaining three cases exhibited a predominantly solid morphology, characterized by dense fibroblastic-myofibroblastic stroma and rare myxoid degenerative areas containing spindled and bipolar cells. Scant chronic inflammation was observed in the majority of cases.

**Figure 1:**
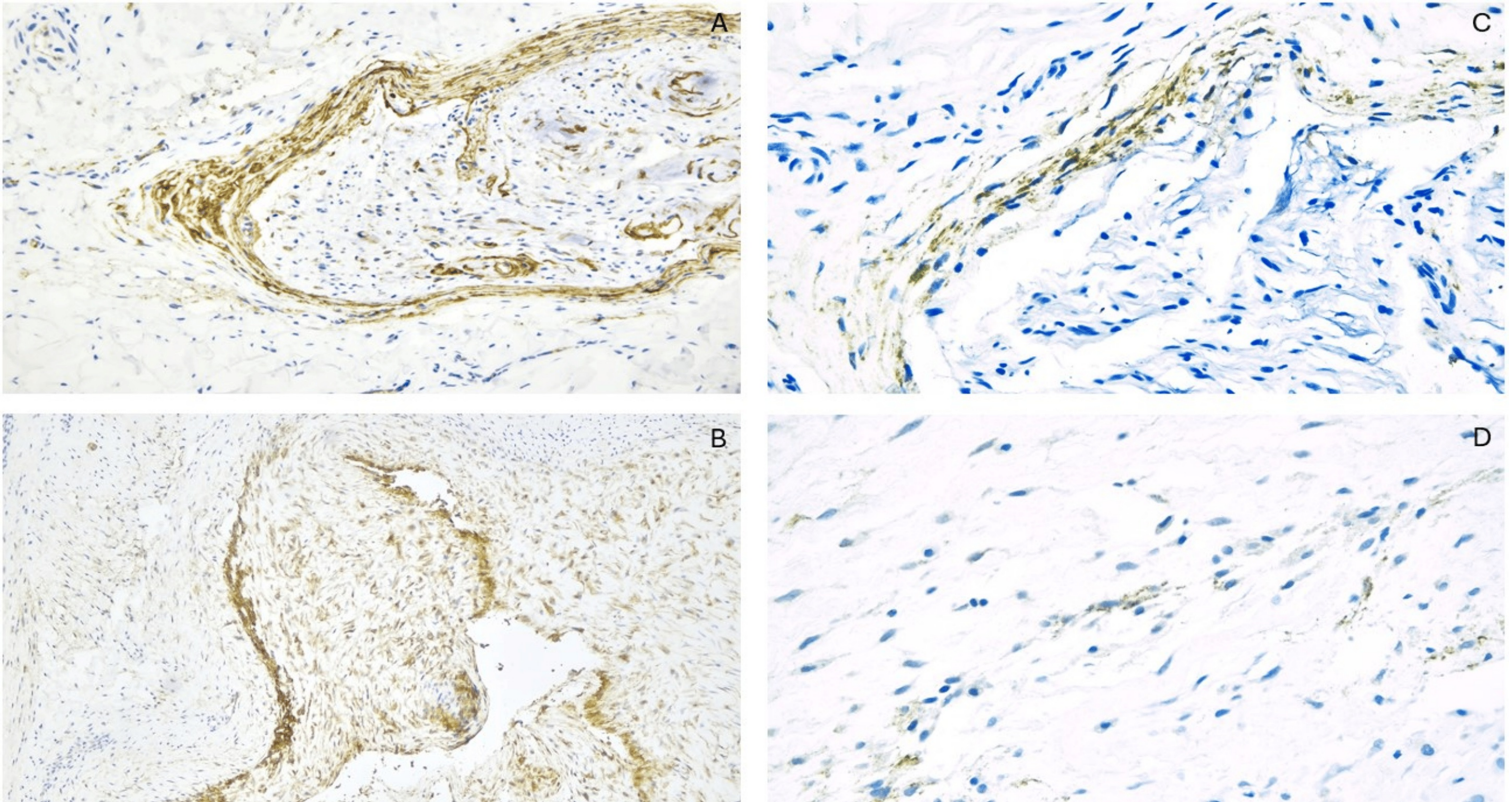
(A) GLUT1, 20X, shows positive staining in an entrapped nerve within a ganglion cyst excision specimen, compared to the GLUT1 staining pattern observed in the cellular components of the ganglion cyst (B, GLUT1, 10X). The EMA staining pattern is demonstrated in the entrapped nerve (C, 20X) and stromal cells of the ganglion cyst excision specimen (D, 20X). EMA, epithelial membrane antigen; GLUT1, glucose transporter 1

**Figure 2:**
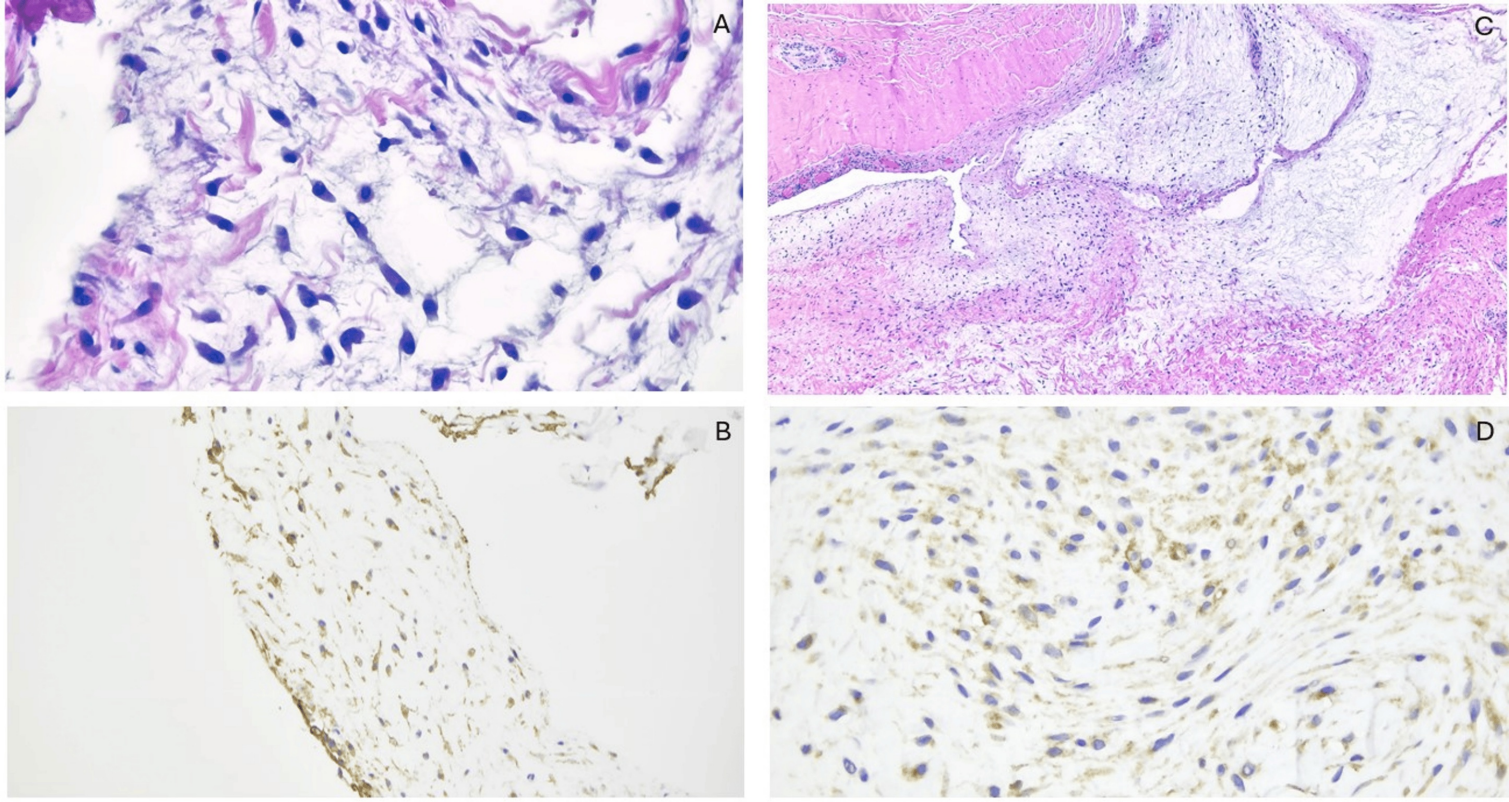
(A, B) Histology of the core biopsy from a ganglion cyst misdiagnosed as a perineurioma, showing bland bipolar spindle-shaped cells in a loose fibromyxoid stroma (H&E, 20X) and immunoreactivity for GLUT1 (4X). (C, D) The excisional specimen of the same lesion reveals cystic changes, lobules of fibromyxoid stroma, and thickened bands of collagenous hyalinized septae (H&E, 4X). The stromal cells again exhibit cytoplasmic staining with GLUT1 (20X). GLUT1, glucose transporter 1

GLUT1 staining demonstrated a membranous pattern in all cases, with occasional cytoplasmic expression (Figure 1A-1D). Intense staining was observed in stellate cells within the myxoid matrix background. EMA showed a membranous expression that was focal to diffuse in 12 cases (85.71%), while it was negative in two cases (14.29%) (Figure 2A-2D).

Table 1 shows the age, gender, lesion site, size, and staining results for GLUT1 and EMA in each case analyzed.

**Table 1:** Summary of 14 cases from the current study, including age, gender, location, size, and immunoreactivity for GLUT1 and EMA All lesional cells in the cases were positive for GLUT1. EMA, epithelial membrane antigen; GLUT1, glucose transporter 1

Case #	Age (Y)	Gender	Location	Size (cm)	GLUT1	EMA
1	76	M	Right wrist	2.3	+	+
2	89	M	Left thumb	1.6	+	+
3	30	F	Right ring finger	0.7	+	+
4	36	F	Right middle finger	0.9	+	+
5	20	F	Right wrist	0.9	+	+
6	64	F	Left dorsal wrist	1.5	+	+
7	58	F	Left volar wrist	1.1	+	+
8	45	F	Left dorsal wrist	1.4	+	Negative
9	63	F	Left dorsal wrist	0.9	+	+
10	36	F	Left hand	1	+	+
11	71	M	Left middle finger	0.3	+	Negative
12	69	F	Left finger	1.2	+	+
13	21	F	Right lateral knee	1.1	+	+
14	74	F	Left finger	0.6	+	+

## Discussion

In this study, we further explore the non-specific expression of GLUT1 immunohistochemical staining in ganglion cysts. Although the diagnosis of ganglion cysts is usually straightforward and rarely requires ancillary testing, the distinction between ganglion cysts and perineuriomas can be difficult in limited biopsy specimens, as both lesions share many overlapping features. This overlap extends beyond morphology to include the expression of both EMA and GLUT1. This raises the possibility that ganglion cysts could represent cystic degeneration of localized hyperplastic perineurial cells, which may proliferate secondary to irritation or injury. This hypothesis has yet to be explored, and further research is needed to confirm or refute it.

GLUT1 is a major transporter that regulates glucose distribution and movement. It is primarily expressed on erythrocytes, endothelial cells in the blood-brain barrier, muscle cells, and neurons at variable concentrations. Several tumors, including colorectal carcinoma and pulmonary carcinomas, also express GLUT1 [8]. In neoplastic processes, increased metabolism leads to elevated lactic fermentation and increased glucose uptake, which is largely mediated by glucose transport proteins encoded by the SLC2A1 gene [9].

GLUT1 was first described by North et al. as a marker useful for differentiating juvenile hemangioma (which expresses GLUT1) from other vascular lesions such as vascular malformations, pyogenic granuloma, Kaposiform hemangioendothelioma, and epithelioid hemangioendothelioma (which do not express GLUT1) [10]. Additionally, GLUT1 is a sensitive immunohistochemical marker for perineural cells and a subset of mesenchymal neoplasms, including chordoma, desmoplastic small round cell tumors, desmoid-type fibromatosis, epithelioid sarcoma, gastrointestinal stromal tumor, myoepithelioma, schwannoma, and undifferentiated pleomorphic sarcoma [5,11]. However, caution is advised when interpreting GLUT1 immunoreactivity in core biopsies of soft tissue lesions with cystic and fibromyxoid histology, as GLUT1 positivity is not specific. The differential diagnosis for such lesions includes benign and low-grade myxoid neoplasms. Therefore, it is important to correlate immunohistochemical findings with clinical and radiological data to avoid misinterpretation.

Perineuriomas are rare benign neoplasms that arise from myofibroblasts forming the peripheral nerve perineurium [12]. These tumors may present as intraneural or extraneural (soft tissue) subtypes. The extraneural subtypes include reticular, sclerosing, and soft tissue forms, and they typically present as asymptomatic, solitary nodules or papules on the extremities of adults [13]. Histologically, perineuriomas are characterized by concentric arrangements of perineurial cells around nerve bulbs (“pseudo-onion bulb” formation), along with whorling and fascicular growth patterns. The tumor cells may infiltrate the surrounding stroma, and alternating hypocellular and hypercellular areas with focal myxoid features are common. Perineuriomas are typically immunoreactive for EMA, GLUT1, CD34, and claudin-1 [3,14,15].

In contrast, ganglion cysts are relatively common benign soft tissue lesions, most commonly seen in the wrist and hand. Although the exact etiology is unclear, ganglion cysts likely arise due to cystic and myxoid degeneration and loosening of fibro-connective tendinous stroma, without a definitive epithelial lining or connection to the joint cavity [16,17]. Ganglion cysts may be focally lined by cells with myofibroblastic or mesenchymal features, distinct from the synovial lining seen in synovial cysts [18]. Ganglion cysts are nonneoplastic lesions that can occur both intraneurally and extraneurally. Intraneural ganglion cysts may express GLUT1 and EMA due to entrapped perineurial cells [19]. In the current study, the majority of the cases were extraneural subtypes, all of which expressed GLUT1, with EMA expression in a subset.

Image-guided core needle biopsy is the preferred modality for obtaining a preliminary diagnosis of soft tissue neoplasms, with lower complication rates and a faster tissue acquisition process. Core biopsies have an approximate 96% concordance rate with final pathology results [20]. However, there is considerable overlap between the histology of myxoid neoplasms and lesions with focal myxoid features, with a wide range of biological behaviors, from benign to recurrent or malignant soft tissue sarcomas. As a result, distinguishing between lesions on core needle biopsy can be challenging. The interpretation of lesions like ganglion cysts and myxoid perineuriomas is particularly difficult, as both share fibromyxoid features, as demonstrated in this study. Immunohistochemical stains alone are not sufficient to differentiate between ganglion cysts and perineuriomas, underscoring the need for correlation with clinical and radiological information when diagnosing lesions based on needle core biopsies.

In the present study, spindle-shaped cells in ganglion cysts from both core biopsies and excision specimens were positive for GLUT1 and, in some cases, also positive for EMA. One of the sentinel cases was a solid knee mass that was initially diagnosed as a perineurioma based on biopsy morphology and immunoreactivity to GLUT1 and EMA. However, examination of the excision specimen revealed that the morphology was more consistent with a ganglion cyst with focal cystic and myxoid degeneration. Given that both intraneural and extraneural forms of perineuriomas and ganglion cysts have been described, significant overlap in the morphological and immunohistochemical features of these lesions is possible.

A major limitation of this study was the small size of the core biopsy specimens, which hindered the definitive classification of the lesion. Excision specimens were essential for accurate diagnosis, and in some cases, more advanced techniques, such as molecular studies, may be required for further investigation of these lesions. Immunohistochemical stains alone were insufficient to differentiate between ganglion cysts and perineuriomas, highlighting the importance of integrating clinical and radiological data when diagnosing lesions based on core needle biopsy.

## Conclusions

GLUT1 immunoreactivity in ganglion cysts is likely not useful for differentiating them from other benign and low-grade myxoid neoplasms with fibromyxoid morphology on core biopsy specimens. Moreover, the coexpression of GLUT1 and EMA in both perineuriomas and ganglion cysts may indicate a shared histogenetic origin for these lesions. Therefore, clinical and radiographic findings are crucial for establishing the key distinctions in soft tissue neoplasms that are rich in myxoid matrix.
